# High Expression of Neuro-Oncological Ventral Antigen 1 Correlates with Poor Prognosis in Hepatocellular Carcinoma

**DOI:** 10.1371/journal.pone.0090955

**Published:** 2014-03-07

**Authors:** Yi-An Zhang, Ji-Min Zhu, Jie Yin, Wen-Qing Tang, Yan-Mei Guo, Xi-Zhong Shen, Tao-Tao Liu

**Affiliations:** 1 Department of Gastroenterology, Zhongshan Hospital of Fudan University, Shanghai, China; 2 Department of Gastroenterology, Tenth People’s Hospital of Tongji University, Shanghai, China; The University of Hong Kong, China

## Abstract

Neuro-oncological ventral antigen 1 (Nova1) is a neuron-specific RNA-binding protein in human paraneoplastic opsoclonus-myoclonus ataxia accompanying with malignant tumors, but its role in hepatocellular carcinoma (HCC) remains elusive. In this study, we found that overexpressed intratumoral Nova1 was associated with poor survival rate and increased recurrence rate of HCC, especially early recurrence, and was an independent prognostic factor for overall survival rate and tumor recurrence. HCC cell lines over-expressing Nova1 exhibited greater potentials in cell proliferation, invasion and migration, while knockdown of Nova1 had the opposite effects. All these findings indicate that Nova1 may act as a prognostic marker for poor outcome and high recurrence in HCC.

## Introduction

Hepatocellular carcinoma (HCC) represents the third cause of cancer-related death worldwide [1]. Although surgical resection improves survival rate of early-stage HCC patients, the high recurrence rate still exists. So far, alpha-fetoprotein (AFP) is the most common marker used for early detection and follow-up of patients during treatment [2]. However, due to existing AFP-negative HCC patients, new biomarkers that predict early recurrence and metastasis of HCC still need to be discovered [3].

Neuro-oncological ventral antigen 1 (Nova1) was first identified as a neuron-specific RNA binding protein in paraneoplastic opsoclonus-myoclonus ataxia (POMA), which is associated with breast cancer, fallopian cancer and small cell lung cancer [4], [5]. The aberrant expression of Nova1 in tumor cells interferes the RNA-binding activity and triggers host immune response, leading to the development of POMA [6]. Further, autoantibodies against Nova1 were found in the serum and/or cerebrospinal fluid of patients before the cancer becomes symptomatic, which implies that these antibodies may have utility for early detection of these disorders [7]. Unfortunately, Nova1 expression in HCC has not yet been observed.

In this study, Nova1 was identified in both tumor and paired peritumoral tissues of 91 HCC patients. Survival analysis indicated that high expression of intratumoral Nova1 was associated with poor prognosis after HCC curative resection. Later, the existence of Nova1 was confirmed in HCC tissues, as well as HCC cell lines and one normal liver cell, and then a series of *in vitro* experiments were performed to investigate the function of Nova1 for cell proliferation, invasion, and migration ability in HCC cell lines. Taken together, all these data support the hypothesis that Nova1 plays a role in HCC developments and may serve as a prognostic marker for tumor relapse and progression.

## Materials and Methods

### Patients and Specimens

Ninety-one HCC patients underwent curative resection were enrolled in this study. Tumor and peritumoral specimens were collected from HCC patients who underwent curative resection in Liver Cancer Institute, Zhongshan Hospital of Fudan University (from October 1, 2006 to December 31, 2008). Clinical characteristics of all patients were summarized (Table 1). HCC was diagnosed by histology, a tumor found by ultrasound, or other diagnostic imaging methods with an AFP level greater than 400 ng/mL [8]. None of these patients took anticancer therapies before surgery. HCC and their adjacent non-cancerous tissues from four patients were selected randomly from 91 HCC patients for Western blotting.

**Table 1:** Characteristics of 91 HCC patients.

characteristics		peritumoral Nova1			intratumoral Nova1		
		Low (n = 32)	High (n = 59)	*p*	Low (n = 20)	High (n = 71)	*p*
Gender	Female	5	8	0.788	5	8	0.121
	Male	27	51		15	63	
Age (years)	≤53	22	29	0.072	8	43	0.102
	>53	10	30		12	28	
ALT (U/L)	≤75	13	35	0.088	11	38	0.907
	>75	19	24		9	33	
Preoperative AFP (ng/mL)	≤20	3	9	0.530*	2	10	1.000*
	>20	29	50		18	61	
HBsAg	Negative	3	5	1.000*	2	6	1.000*
	Positive	29	54		18	65	
Cirrhosis	No	4	5	0.715*	2	7	1.000*
	Yes	28	54		18	64	
Vascular invasion	No	21	30	0.175	14	37	0.155
	Yes	11	29		6	34	
Tumor Number	Single	17	37	0.374	8	46	0.046
	Multiple	15	22		12	25	
Tumor Size (cm)	≤5	17	29	0.717	13	32	0.115
	>5	15	30		7	39	
Encapsulation	No	22	36	0.464	14	44	0.509
	Yes	10	23		6	27	
Tumor Differentiation	I–II	22	42	0.808	14	50	0.971
	III–IV	10	17		6	21	
TNM stage	I	15	27	0.919	7	35	0.257
	II–III	17	32		13	36	
BCLC stage	A	9	10	0.21	7	12	0.079
	B/C	23	49		13	59	

*Fisher’s exact test; χ^2^ for all other analyses. Abbreviations: HbsAg, hepatitis B surface antigen; ALT, alanine aminotransferase. TNM, tumor-node-metastasis.

Follow-up was finished until December 31, 2011. All patients were followed every 3 months. The median follow-up was 40.9 months (ranging from 2.3 to 62.1 months). Serum AFP, abdomen ultrasonography, chest X-ray, and CT/MRI were checked in all patients to verify cancer recurrence. Diagnosis of cancer recurrence was based on typical imaging appearance in CT/MRI scan and elevated AFP. Common reasons of decease in the present study included cancer recurrence, distal metastasis and complicated liver cirrhosis.

Overall survival rate (OS) was defined as intervals from the date of surgery to decease or the last observation. Time to recurrence rate (TTR) was measured from the date of resection to either detection of recurrent tumor or the last follow-up assessment. Clinical stages of tumor were determined according to the TNM classification system of International Union Against Cancer [9]. The histologic grade of tumor differentiation was assigned by Edmondson-Steiner classification [10].

The study was approved by the Ethics Review Committee, Zhongshan Hospital of Fudan University. All patients provided written consent forms for this study.

### Cells Culture and Reagents

Four human HCC and one normal liver cell lines were used in this study. SMMC-7721 and L02 cells were purchased from Shanghai Institute of Cell Biology, Chinese Academy of Sciences (Shanghai, China). Huh7 cell was from Riken Cell Bank (Tsukuba Science City, Japan). MHCC-97H and MHCC-97L cells were from Liver Cancer Institute, Zhongshan Hospital of Fudan University (Shanghai, China) [11]. SMMC-7721 and L02 cells were cultured in RPMI medium 1640 (GIBCO, USA). Huh7, MHCC-97H and MHCC-97L cells were cultured in DMEM supplemented with 10% fetal bovine serum (GIBCO, Austria). Cells were cultured in 37°C incubator with humidified atmosphere containing 5% CO_2_. Goat anti-human Nova1 polyclonal antibody was purchased from LifeSpan Biosciences (Seattle, USA) for detecting Nova1 in HCC tissues and from Abcam (Cambridge, USA) for detecting Nova1 in cell lines. HRP-conjugated rabbit anti-goat antibody was purchased from EarthOx, LLC (San Francisco, USA). Mouse anti-GAPDH antibody, HRP-conjugated goat anti-mouse antibody and HRP-conjugated goat anti-rabbit antibody were purchased from Beyotime Institute of Biotechnology (Shanghai, China). The enhanced chemiluminescence Western blotting Substrate System was purchased from Pierce (Rockford, USA).

### Establishment of Cell Lines to Induce or Silence Nova1 Expression

The target sequence of small hairpin RNA (shRNA) for Nova1 is 5′-AGCAC AGCAG GTCTG ATAAT AG-3′. A lentiviral vector-expressing shRNA specific to Nova1 was subcloned into pSLIK (a single lentivector for inducible knockdown) with a tetracycline response element (TRE), and then cotransfected with pCMVR-delta 8.9 and VSVG packaging vector into 293FT by FuGEN HD (Roche, USA). 48 hours after transfection, medium with lentivirus were infected into Huh7 cells for 72 hours. Lentiviral vector containing either human Nova1 cDNA with green fluorencesence protein VENUS or a specific shRNA, together with two packaging vectors were cotransfected to SMMC-7721 or Huh7. Polyclonal cells were then selected, cloned, and screened for overexpression or knockdown of Nova1. Doxycycline (Dox) was used as an inducer to switch the targeted gene expression.

### Immunochemical Staining

Five-micron thick sections were fixed in 4% formaldehyde and embedded in paraffin. Tissue samples were deparaffinized and rehydrated, followed by high-temperature antigen retrieval via microwave in 0.1 M citrate solution (pH 6.0) for 15 min. After blocked with 5% normal goat serum at room temperature for 30 min, the sections were incubated with goat anti-human Nova1 antibody (LifeSpan, USA) at 4°C overnight, and then incubated with rabbit anti-goat secondary antibody (EarthOx, USA) at room temperature for 30 min, and finally immunostained by avidin-biotin complex technique using 3,3′-diaminobenzidine. Hematoxylin was used as a counterstain.

The total amount of positive cells in each section was evaluated by two independent investigators masked to clinical outcome and clinicopathologic data. Positive staining cells were observed by using one light microscope (Olympus BX51, Japan). The Friedrichs scoring system was applied to analyze expression of Nova1 in tumor and peritumoral tissues. The intensity of Nova1 staining was divided into four grades, score “0” for negative, “1” for light yellow, “2” for deep yellow, “3” for brown. Nova1 staining positive cells was further subgrouped into score “0” for less than 5%, “1” for 6–20%, “2” for 21–50% and “3” for more than 51%. The total score was defined as staining intensity and scales. Total score higher than 2 is considered as high expression, while under 2 as low expression.

### Real-time Quantitative PCR (qPCR)

A qPCR experiment was performed with SYBR reagent (Takara, Dalian, China) and by ABI prism 7500 sequence detector (Applied biosystems, Japan). The mRNA quantity of specific genes, calculated using the 2^−ΔΔCt^ method, was normalized against β-actin. All the measurements were performed in triplicate. The sequences of the primer pairs were as follows: Nova1-F: 5′-TGCCA TCTTC CCCAA CTACC A-3′, Nova1-R: 5′-TCTCC ACTCA CAGTG ACAAC CCT-3′, β-actin-F: 5′-CATTG CCGAC AGGAT GCA-3′, β-actin-R: 5′-GCCGA TCCAC ACGGA GTACT-3′.

### Western Blotting

Total cell or tissue lysates were generated and equal amount protein was subjected to 10% SDS-PAGE gel, and then transferred onto polyvinylidene difluoride (PVDF) membranes (Millipore, Bedford, USA). Membranes were blocked in blocking solution (50 mM Tri-HCl, 150 mM NaCl, 5% (w/v) non-fat dry milk and 0.1% Tween-20) at room temperature for 1 hour, followed by incubation with Nova1 goat anti-human antibody at 4°C overnight. After three times washing by 0.1% TBS-Tween20, the membrane was incubated with secondary antibody at room temperature for 1.5 h, the blot were demonstrated by enzyme-linked chemiluminescence using a Fluor Chem FC2 chemilumilescent, fluorescent and visible light gel imaging system (Alpha Inotech, USA).

### Proliferation Assay

Cells were plated in a 96-well plate at a concentration of 2000 cells per well. All assays were performed in quadruplicate. At 1, 2, 3, 4 and 5 days, the cell proliferation assay was performed by addition of 10 µL cell counting kit 8 (CCK8) solution (Beyotime Institute of Biotechnology, Shanghai, China) to each well, followed by incubation at 37°C for 2 h. Absorbance was measured at a wavelength of 450 nm using a microplate reader (Flexstation III ROM V2.1.28, USA).

### Scratch-wound Assay

Cells at 100% confluence in six-well plates were scraped with a sterile micropipette tip to create a denuded area of constant width of 1 mm. Cells were washed with PBS solution to remove cell debris, and then cultured in serum free medium. The wound closure was monitored and photographed at 48 h after wounding.

### Transwell Migration and Invasion Assay

Cells were harvested and suspended in serum free medium supplemented with 1% BSA. For transwell migration assay, cell suspension was loaded into the top chamber with a non-coated membrane at a concentration of 1×10^5^ cells per 100 µL. For transwell invasion assays, cell suspensions were loaded into the top chamber with a Matrige-coated membrane at a concentration of 5×10^5^ cells per 100 µL. In both assays, medium containing 20% FBS was used as a chemoattractant in the lower chamber. After incubation for 48 h, cells that had migrated or invaded to the lower surface of the filter were fixed with 4% paraformaldehyde, followed by stained with Giemsa. Cells were counted in four independent fields with light microscope.

### Statistical Analysis

All values were expressed as mean ± standard deviation (SD). All statistical analyses were performed using the SPSS 18.0 (SPSS Inc., Chicago, IL). χ^2^-test or Fisher’s exact tests was used for the association of Nova1 expression with the clinicopathologic features. Student’s *t* test and independent sample *t* test were used for comparison between groups. Cumulative survival time was calculated by Kaplan-Meier method and analyzed by the log-rank test. Univariate and multivariate analyses were based on the Cox proportional hazard regression model. *P*<0.05 was considered as statistically significant.

## Results

### Higher Nova1 Expression in HCC Patients Indicates Poor Prognosis

**Figure 1:**
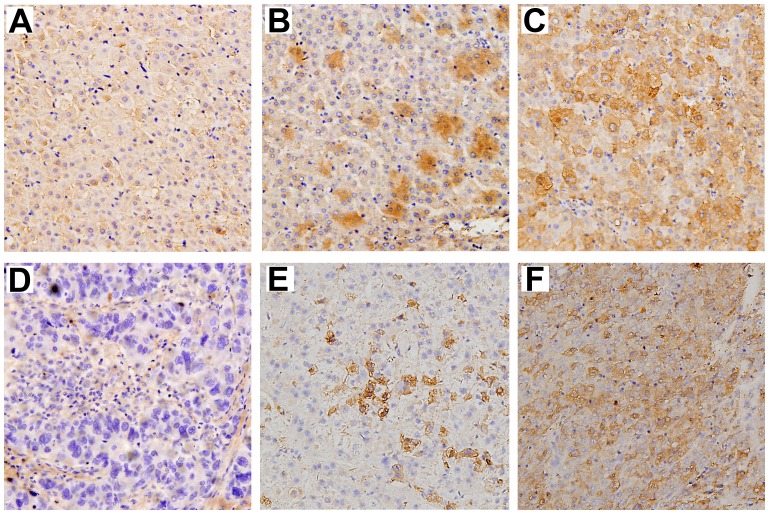
Representative immunohistochemistry staining of Nova1 in tumor and peritumoral tissues of HCC patients. Negative staining (A), moderate staining (B) and intense staining (C) of peritumoral tissues. Negative staining (D), moderate staining (E) and intense staining (F) of tumor tissues. Original magnification: ×200.

To investigate the role of Nova1 in HCC, expression of Nova1 in tumor and paired peritumoral tissues of patients was first determined. Nova1 was focally expressed in cytoplasm of both peritumoral and tumor cells (Fig. 1). The difference of the percentage of Nova1 positive cell in the cancerous and para-cancerous tissues was borderline statistical significance (78.02% (71/91) vs. 64.84% (59/91), respectively, *P = *0.049). Univariate analysis showed that clinical factors including encapsulation, vascular invasion, tumor size, tumor number, TNM stage and BCLC stage, had prognostic significance for both OS and TTR (Table 2). Of note, Intratumoral Nova1 was an unfavorable predictor for OS and TTR (HR = 2.210 and 2.543, respectively, *P* < 0.01; Table 2). Multivariate Cox analysis showed that vascular invasion, tumor size, TNM stage and intratumoral Nova1 was significantly associated with OS (*P = *0.036, 0.044, 0.013, and 0.025, respectively), while tumor size, tumor number and intratumoral Nova1 levels was similarly associated with TTR (*P* = 0.007 and 0.002, respectively; Table 3).

**Table 2:** Univariate analysis of factors associated with survival rate and recurrence rate.

Variables	OS		TTR	
	Hazard ratio (95% CI)	*P*	Hazard ratio (95% CI)	*P*
Sex (male vs. female)	1.091 (0.462–2.574)	0.483	1.081 (0.512–2.283)	0.839
Age, y (>53 vs. ≤53)	0.993 (0.968–1.018)	0.586	0.993 (0.971–1.015)	0.514
Preoperative AFP, ng/mL (>20 vs. ≤20)	0.999 (0.993–1.005)	0.763	1.154 (0.460–2.897)	0.76
ALT, U/L (>75 vs. ≤75)	1.744 (0.963–3.158)	0.066	1.208 (0.709–2.059)	0.486
HBsAg (negative vs. positive)	0.705 (0.278–1.786)	0.461	0.890 (0.355–2.233)	0.804
Liver cirrhosis (yes vs. no)	1.997 (0.619–6.444)	0.247	1.555 (0.621–3.895)	0.346
Encapsulation (yes vs. no)	0.538 (0.282–1.023)	0.059	0.520 (0.297–0.909)	**0.022**
Vascular invasion	2.537 (1.411–4.562)	**0.002**	1.907 (1.133–3.211)	**0.015**
Tumor size, cm (>5 vs. ≤5)	2.506 (1.362–4.611)	**0.003**	2.742 (1.601–4.698)	**<0.001**
Tumor number (multiple vs. single)	2.201 (1.230–3.939)	**0.008**	2.089 (1.241–3.518)	**0.006**
Tumor differentiation (I–II vs. III–IV)	1.067 (0.568–2.007)	0.84	1.081 (0.620–1.885)	0.783
TNM stage (II–III vs. I)	1.693 (1.269–2.259)	**<0.001**	2.452 (1.426–4.214)	**0.001**
BCLC stage (B/C vs. A)	3.648 (1.305–10.119)	**0.014**	2.731 (1.371–5.439)	**0.004**
Intratumoral Nova1 (High vs. Low)	2.210 (1.207–4.084)	**0.008**	2.543 (1.089–3.084)	**0.002**
Paraneoplastic Nova1 (High vs. Low)	1.018 (0.566–1.831)	0.953	1.089(0.643–1.843)	0.751

Bold items have been considered statsitically significant. Univariate analysis was based on the Cox proportional hazard regression model. Abbreviations: 95% CI, 95% confidence interval; OS, overall survival rate; TTR, time to recurrence rate; HbsAg, hepatitis B surface antigen; ALT, alanine aminotransferase; TNM, tumor-node-metastasis.

**Table 3:** Multivariate analysis of factors associated with OS and TTR.

	Hazard ratio (95% CI)	*P*	
**OS**			
Vascular invasion	1.995 (1.045–3.806)	**0.036**	
Tumor size, cm (>5 vs. ≤5)	1.941 (1.017–3.704)	**0.044**	
Tumor number (multiple vs. single)		NS	
TNM stage (II–III vs. I)	2.314 (1.192–4.492)	**0.013**	
BCLC stage (B/C vs. A)		NS	
Intratumoral Nova1 (negative vs. positive)	2.120 (1.101–4.080)	**0.025**	
**TTR**			
Encapsulation (yes vs.no)		NS	
Vascular invasion		NS	
Tumor size, cm (>5 vs. ≤5)	2.905 (1.628–5.184)	**<0.001**	
Tumor number (multiple vs. single)	2.168 (1.239–3.793)	**0.007**	
TNM stage (II–III vs. I)		NS	
BCLC stage (B/C vs. A)		NS	
Intratumoral Nova1 (negative vs. positive)	2.403 (1.395–4.250)	**0.002**	

Bold items have been considered statistically significant. Multivariate analysis and Cox proportional hazards regression model were used. Variables were adopted for their prognostic significance by univariate analysis (*P* < 0.05). NS, not significant, OS, overall survival rate; TTR, time to recurrence rate.

Although Nova1 was an unfavorable predictor for cancer recurrence, the relationship between Nova1 expression and early recurrence (defined as cancer relapses within 24 months) was further analyzed. Importantly, intratumoral Nova1 presented a strong relationship with early recurrence (*P = *0.012; Table 4). In addition, vascular invasion, tumor size and tumor number also showed predictive power in determining HCC early recurrence (*P = *0.008, 0.002 and 0.001, respectively).

**Table 4:** Prognostic factors for early recurrence.

Factor	Early recurrence		
	Univariate *P*	Multivariate HR (95%CI)	*P*
Encapsulation (yes vs.no)	0.018		NS
Vascular invasion	0.001	2.284 (1.236–4.220)	**0.008**
Tumor size, cm (>5 vs.≤5)	<0.001	2.579 (1.419–4.687)	**0.002**
Tumor number (multiple vs. single)	0.011	2.780 (1.540–5.018)	**0.001**
TNM stage (II-III vs. I)	0.003		NS
BCLC stage (A vs. B/C)	0.003		NS
Intratumoral Nova1 (negative vs. positive)	0.012	2.163 (1.183–3.953)	**0.012**

Bold items have been considered statsitically significant. Univeriate and multivariate analysis were performed using Cox proportional hazards regression model. Abbreviations: HR, Hazard Ratio; 95% CI, 95% confidence interval; TNM, tumor-node-metastasis; NS, not significant.

**Figure 2:**
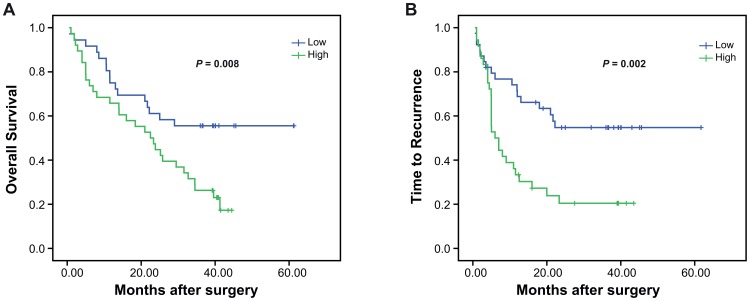
High expression of tumor Nova1 correlates with poor survival rate. Overall survival (A) and time to recurrence (B) between patients with high and low expression of Nova1 were estimated by the Kaplan-Meier method and compared by the log rank test.

Kaplan-Meier analysis and the log-rank test were then performed to evaluate the effects of Nova1 on survival rate (Fig. 2). Patients with higher intratumoral Nova1 had shorter OS rate (median, 27.27 months) and less TTR time (median, 13.50 months), while its peritumoral level were not associated with OS or TTR (Data not shown). These results demonstrated that Nova1 expression could be a predicting marker for OS and TTR of HCC patients.

### Upregulated Nova1 is Detected in Both HCC Tissues and Cell Lines

**Figure 3:**
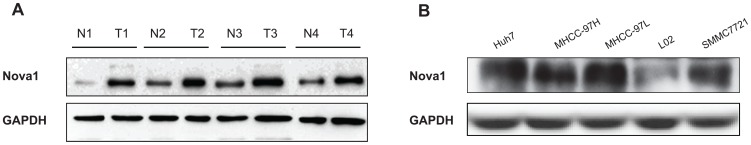
Nova1 expression in HCC tissues, as well as four HCC and one normal liver cell lines. (A) Nova1 expression in four pairs of HCC (T) and paired nontumor tissues (N) of the liver. (B) Nova1 expression in indicated HCC (including MHCC-97H, MHCC-97L, Huh7 and SMMC-7721 cell lines) and one normal liver cell lines.

To reconfirm the hypothesis that Nova1 in HCC tissues is higher than paired peritumoral tissues, Nova1 expression in four pairs of tumor and adjacent peritumoral tissues of HCC patients was measured. Nova1 was presented both in tumor and peritumoral tissues, but much higher in tumor tissues (Fig. 3A). Next Nova1 was further measured in four HCC cell lines with different metastatic potentials and one normal liver cell line as a control. Nova1 was much upregulated in the most invasive HCC cells (MHCC-97H and -97L) and Huh7 cells than in either less invasive SMMC-7721 cells or normal hepatocytes (L02) (Fig. 3B), suggesting that its expression may relate to malignant potential of HCC.

### Dox Manipulates Nova1 Expression in a Dose- and time-dependent Manner

**Figure 4:**
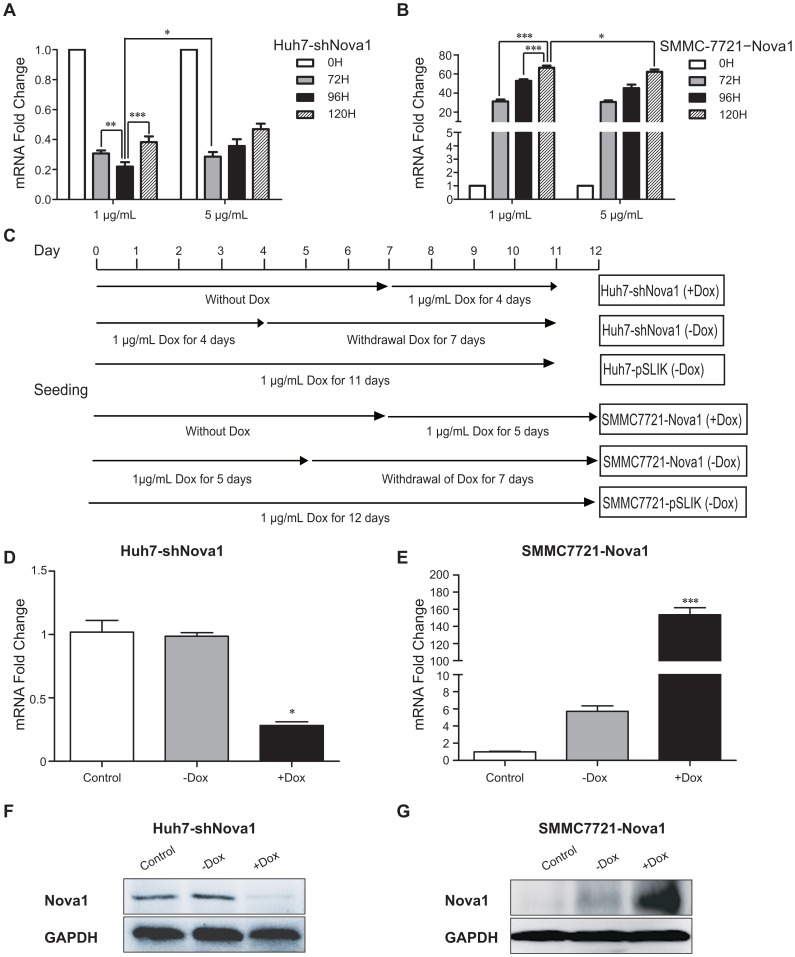
Doxycycline manipulates Nova1 expression in a dose- and time-dependent manner. (A) Time course of mRNA level of Nova1 using 1 µg/mL and 5 µg/mL Dox treatment in Huh7-shNova1 cells. (B) Time course of mRNA level of Nova1 using 1 µg/mL and 5 µg/mL Dox treatment in SMMC-7721−Nova1 cells. (C) Experimental protocol for Dox treatment in Huh7-shNova1 (+Dox), Huh7-shNova1 (−Dox) and Huh7-pSLIK, as well as in SMMC-7721−Nova1 (+Dox), SMMC-7721−Nova1 (−Dox) and SMMC-7721−pSLIK cells. (D) The mRNA level of Nova1 in Huh7-shNova1 and Huh7-pSLIK cells treated with 1 µg/mL Dox according to experimental protocol shown in Fig. 4C. (E) The mRNA level of Nova1 in SMMC-7721−Nova1 and SMMC-7721−pSLIK treated with 1 µg/mL Dox according to expreimental protocol shown in Fig. 4C. (F) The protein level of Nova1 in Huh7- shNova1 and Huh7-pSLIK cells treated with 1 µg/mL Dox according to experimental protocol shown in Fig. 4C. (G) The protein level of Nova1 in SMMC-7721−Nova1 and SMMC-7721−pSLIK treated with 1 µg/mL Dox according to expreimental protocol shown in Fig. 4C. β-actin and GAPDH were performed as a loading control in qPCR and Western blotting, respectively. Control cells were cells that carry pSLIK empty vector; +Dox: Dox treatment; -Dox: Dox treatment for indicated times and then withdrawal Dox for another seven days. Huh7-shNova1, monoclonal Huh7 cells with knockdown of Nova1; SMMC-7721−Nova1, monoclonal SMMC-7721 cells with overexpression of Nova1. *, *P*<0.05; **, *P*<0.01; ***, *P*<0.001.

Based on results above, we found that the ability of HCC invasion is positively correlated with the Nova1 expression. To further test this, Huh7 and SMMC-7721 cells were chosen to establish the inducible system to overexpress or knockdown Nova1. The pSLIK vector system was used to generate stable Dox-inducible Nova1 expression in the two HCC cells. The mRNA level of Nova1 upon Dox treatment with different concentration and exposure time in the two cell lines was then examined. The knockdown ratio of Nova1 for Huh7 cells reached highest under 1 µg/mL Dox treatment at 96 exposure hours (Fig. 4A), while the overexpression ratio of Nova1 for SMMC-7721 cells reached highest under 1 µg/mL Dox treatment at 120 exposure hours (Fig. 4B). However, both the knockdown and overexpression experiments did not change in parallel with higher Dox concentration (5 µg/mL), we reasoned that high concentration of Dox suppressed or silenced the gene expression. Thus, 1 µg/mL Dox was identified as the most efficient concentration for use in this work, which is in agreement with previous studies [12]–[14].

Next, we divided the Huh7 cells into three groups: 1 µg/mL Dox treatment for four days; 1 µg/mL Dox treatment for four days, then withdrawal Dox for another 7 days; Huh7 cells that transfected with pSLIK empty vectors treated with 1 µg/mL Dox to exclude that the effects observed are not due to Dox for 11 days. Further, we divided the SMMC-7721 cells into three groups: 1 µg/mL Dox treatment for 5 days; 1 µg/mL Dox treatment for 5 days, then withdrawal Dox for another 7 days; SMMC-7721 cell that transfected with pSLIK empty vectors treated with 1 µg/mL Dox for 12 days (Fig. 4C). The results indicated that in the inducible system at the end of processing, both the mRNA and protein level of Nova1 could be reversibly up-regulated or down-regulated by Dox (Fig. 4, D–G).

### Nova1 Promotes HCC Cell Proliferation

**Figure 5:**
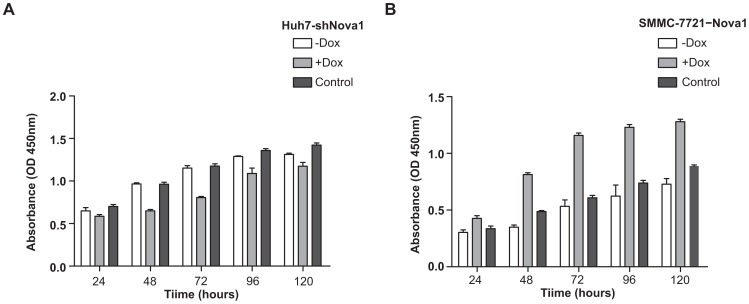
Nova1 promotes HCC cell proliferation. (A) The growth rates of Nova1 knockdown group (Huh7-shNova1 cells treated with Dox for 96 hours), Huh7-shNova1(−Dox) and Huh7-pSLIK cells at indicated time points. (B) The growth rates of Nova1 overexpression group (SMMC-7721−Nova1 cells treated with Dox for 120 hours), SMMC-7721−Nova1(−Dox) and SMMC-7721−pSLIK cells at indicated time points. The growth curves were determined in quadruple, and they were representative of three independent experiments. Control cells were cells that carry pSLIK empty vector; +Dox: Dox treatment; -Dox: Dox treatment for indicated times and then withdrawal Dox for another seven days. Huh7-shNova1, monoclonal Huh7 cells with knockdown of Nova1; SMMC-7721−Nova1, monoclonal SMMC-7721 cells with overexpression of Nova1. *, *P*<0.05; **, *P*<0.01; ***, *P*<0.001.

To demonstrate that Nova1 promotes HCC cell proliferation, cell proliferation potential was studied by the inducible expression system. The growth rate in Huh7-shNova1 cells (+Dox) was significantly reduced as compared with that in Huh7-shNova1 cells (−Dox) and Huh7-pSLIK cells at all indicated time points (*P*<0.05), except the growth rate for comparison of Huh7-shNova1 (−Dox) and Huh7-shNova1 cells (+Dox) cells at 24 h (Fig. 5A). These results indicated that silencing Nova1 expression in Huh7 cells attenuated cell growth rate as compared with the control groups, while withdrawal Dox to repress Nova1 could partially restore the cell proliferation potential. On the contrary, the growth rates in SMMC-7721−Nova1 cells (+Dox) were significantly enhanced as compared with SMMC-7721−Nova1 (−Dox) or SMMC-7721−pSLIK cells at all indicated time points (*P*<0.05) (Fig. 5B). The results indicated that overexpressing Nova1 enhanced proliferative ability in SMMC-7721 cells, while withdrawal Dox led to compromise of the proliferation potential. Further, no statistical significance was noticed between Huh7-pSLIK and Huh7-shNova1 cells (−Dox), as well as SMMC-7721−pSLIK or SMMC-7721−Nova1 (−Dox) cells.

### Nova1 Contributes to the Migration and Invasion in HCC

**Figure 6:**
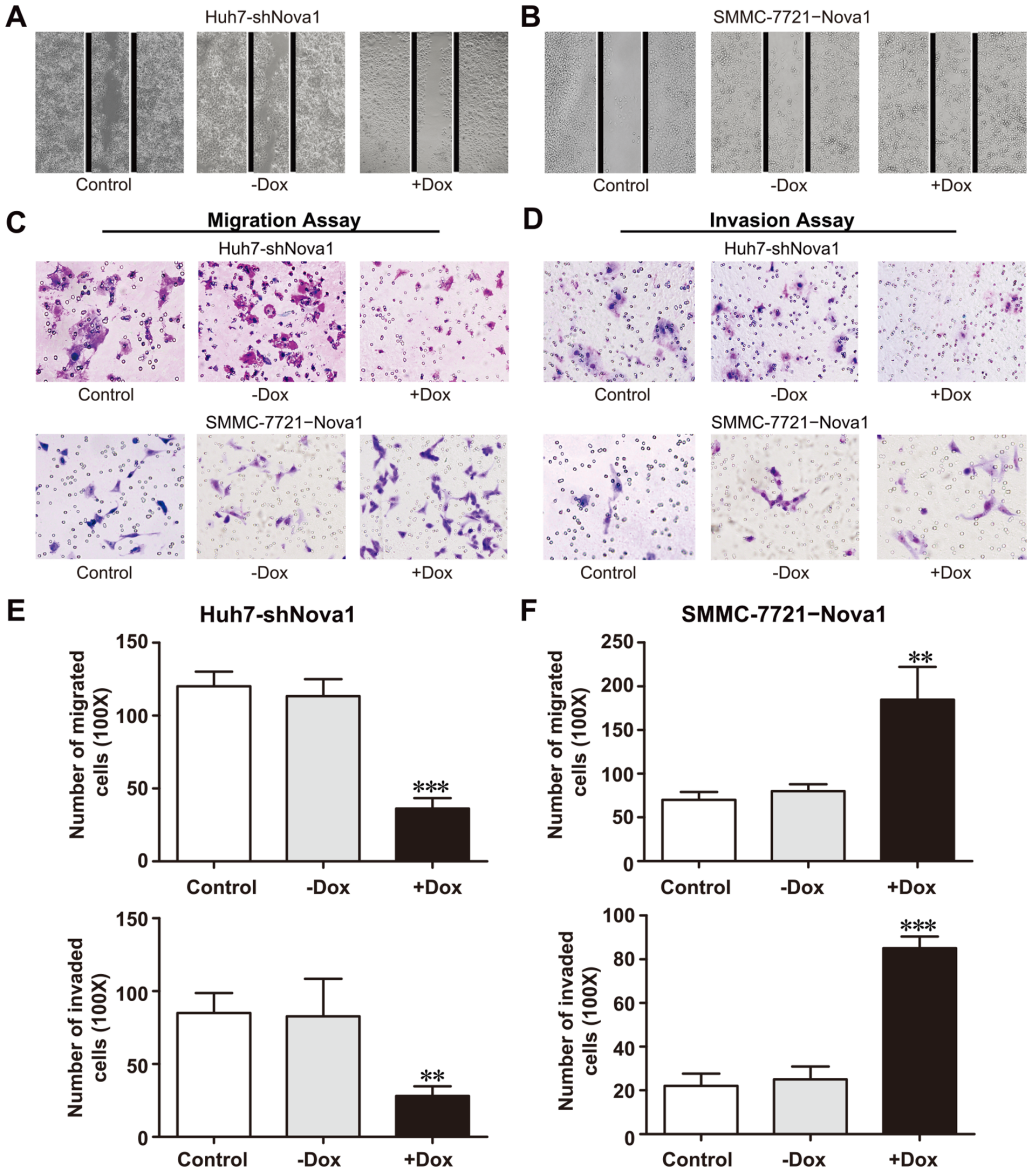
Nova1 promotes cell migration and invasion. (A) Representative images for Huh7-shNova1 (+Dox), Huh7-shNova1 (−Dox) and Huh7-pSLIK cells at 48 h after wounding. (B) Representative images for SMMC-7721−Nova1 (+Dox), SMMC-7721−Nova1 (−Dox) and SMMC-7721−pSLIK cells at 48 h after wounding. (C) Representative images of migration assay for Huh7-shNova1 (+Dox), Huh7-shNova1 (−Dox) and Huh7-pSLIK, as well as SMMC-7721−Nova1 (+Dox), SMMC-7721−Nova1 (−Dox) and SMMC-7721−pSLIK cells (magnification: ×100). (D) Representative images of invasion assay for Huh7-shNova1 (+Dox), Huh7-shNova1 (−Dox) and Huh7-pSLIK, as well as SMMC-7721−Nova1 (+Dox), SMMC-7721−Nova1 (−Dox) and SMMC-7721−pSLIK cells (magnification: ×100). (E) The number of migrated (upper) or invaded (down) cells was manually counted in four independent fields per well with light microscope in Huh7-shNova1 (+Dox), Huh7-shNova1 (−Dox) and Huh7-pSLIK cells. (F) The number of migrated (upper) or invaded (down) cells was manually counted in four independent fields per well with light microscope in SMMC-7721−Nova1 (+Dox), SMMC-7721−Nova1 (−Dox) and SMMC-7721−pSLIK cells. Control cells were cells that carry pSLIK empty vector; +Dox: Dox treatment; -Dox: Dox treatment for indicated times and then withdrawal Dox for another seven days. Huh7-shNova1, monoclonal Huh7 cells with knockdown of Nova1; SMMC-7721−Nova1, monoclonal SMMC-7721 cells with overexpression of Nova1. **, *P*<0.01; ***, *P*<0.001.

Since Nova1 promotes HCC proliferation, we therefore consider the possibility that it also contributes to recurrence and metastasis of HCC. We next examined the migratory and invasive ability of Huh7 and SMMC-7721 cells. The migration of Huh7-shNova1 (+Dox) into the wound area were significantly inhibited as compared with Huh7-shNova1 (−Dox) and Huh7-pSLIK cells after 48 hours (Fig. 6A). The SMMC-7721−Nova1 (+Dox) tended to migrate to the wound area faster and organized a denser cellular network as compared with SMMC-7721−Nova1 (−Dox) and SMMC-7721−pSLIK cells after 48 h (Fig. 6B). The migration of Huh7-shNova1 cells (+Dox) were significantly decreased as compared with Huh7-shNova1 (−Dox) and Huh7-pSLIK cells in the transwell migration assay (Fig. 6C, upper). Likewise, there were fewer invaded cells in Huh7-shNova1 (+Dox) group than in the Huh7−shNova1 (−Dox) and Huh7-pSLIK groups in the invasion assay (Fig. 6D, upper). Consistently, overexpressing Nova1 increased migration and invasion of SMMC-7721−Nova1 cells when treated with Dox (Fig. 6C–D, down). Thus, the findings indicated that overexpression of Nova1 promotes migration and invasive capabilities of HCC cell *in vitro*, and reduction of Nova1 attenuates HCC cell progression.

## Discussion

RNA-binding proteins (RBPs) play key role in post-transcriptional regulation of RNA metabolism, ranging from splicing and editing to transport, localization, and degradation [15]–[17]. Nova1 was the first identified RNA binding protein in human POMA [4], and previously regarded as a specific neuron’s protein [17]. After that, studies recognized it as an onconeural antigen in the antisera of patients with POMA, a motor disorder of brainstem, cerebella, and spinal motor neurons associated with cancer. Unfortunately, to date, there are few case reports and little discussion in the literature addressing the Nova1 autoantibodies expressed in malignant tumors. Brieva-Ruiz *et al.* reported a 71-year-old woman who died from anti-Nova1 positive paraneoplastic cerebellar degeneration associated with breast cancer [18]. Another case reported by Wirtz *et al.* involved a 65-year-old male patient with nausea and vomiting, who was found to have autoantibodies in the serum [19]. Additionally, Stich *et al.* found that the increased concentrations of anti-Nova1 antibody in follow-up serum samples of a breast cancer patient may predict tumor relapse [20].

In the present study, we found that Nova1 is expressed in cytoplasm of both tumor and peritumoral tissues of HCC patients. The difference of the percentage of Nova1 positive cell in the tumor and peritumoral tissues was borderline statistical significant. Interestingly, intratumoral (but not peritumoral) Nova1, correlated to poor survival rate and increased occurrence of cancer relapse. Univariate and multivariate analysis disclosed the relationship of intratumoral Nova1 and OS or TTR in HCC patients that intratumoral Nova1 was an independent prognostic factor for OS and TTR. Moreover, intratumoral Nova1 strongly correlated to HCC early recurrence. However, more HCC patients and longer follow-up study are still required to verify our results in future study.

Likewise, Nova1 was also higher expression in four HCC cell lines than in the normal liver cell line. It was shown that Nova1 is correlated to malignant potential of HCC. Next an inducible expression system manipulated by Dox, either knockdown of Nova1 in Huh7 cell or overexpression of Nova1 in SMMC-7721 cell, was established to study the role of Nova1 in cancer cell behavior. Cell migration and invasion were enhanced in SMMC-7721 cell with overexpression of Nova1, whereas reduced in Huh7 cells with knockdown of Nova1. These results were in accord with the results in our clinical research.

Alternative splicing of pre-mRNAs generate proteins involved in diverse functions. They affect main neurotransmitter receptors localization, binding potential, signal transduction as well as their electrophysiological properties [21], [22]. Ligand-gated ion channels are a group of transmembrane ion channel proteins upon binding to chemical messengers, such as a neurotransmitter. Members of this superfamily include nicotinic acetylcholine receptors, GABA_A_ receptors, glycine, and 5-HT_3_ receptors. It has been reported that these neurotransmitters, as well as their signaling pathways, are involved in carcinogenesis. For example, activation of dopamine D2 receptor inhibits tumor cell proliferation by up-regulating Krupple like factor 4, a negative regulator of the cell cycle in gastric cancer, and through insulin-like growth factor receptor-I and Akt pathway [23]. Increased in GABAergic activity inhibits hepatocellular carcinoma cell growth [24]–[27]. It has been illustrated that Nova1 is a RNA-binding protein, which plays a role for regulating alternative splicing of GABA_A_ receptor γ2, dopamine D2 receptor, and the splice variant α2N of the receptor of glycine [21]. However there is little known about the role of Nova1 for these alternative splicing neurotransmitters in carcinogenesis. Further experiments will be needed to investigate underlying mechanisms of Nova1 in the progression of HCC.

In brief, it is reported in the present study that Nova1 exists in liver cancer cells. High intratumoral Nova1 is associated with poor survival rate and increased cancer recurrence rate. Cell culture experiments *in vitro* demonstrate that Nova1 promotes HCC cells proliferation, migration and invasion. All these study shed a light on the potential diagnostic value of Nova1 in liver cancer development.
